# A novel end-to-end dual-camera system for eye gaze synchrony assessment in face-to-face interaction

**DOI:** 10.3758/s13414-023-02679-4

**Published:** 2023-04-26

**Authors:** Max Thorsson, Martyna A. Galazka, Jakob Åsberg Johnels, Nouchine Hadjikhani

**Affiliations:** 1 Gillberg Neuropsychiatry Centre, Institute of Neuroscience and Physiology, Sahlgrenska Academy, University of Gothenburg, Gothenburg, Sweden; 2 Section of Speech and Language Pathology, Institute of Neuroscience and Physiology, Sahlgrenska Academy, University of Gothenburg, Gothenburg, Sweden; 3 Athinoula A. Martinos Center for Biomedical Imaging, Massachusetts General Hospital, Harvard Medical School, Boston, MA USA

**Keywords:** Face-to-face contact, Dyadic interaction, Eye tracking, Interpersonal synchronization, Deep learning

## Abstract

Quantification of face-to-face interaction can provide highly relevant information in cognitive and psychological science research. Current commercial glint-dependent solutions suffer from several disadvantages and limitations when applied in face-to-face interaction, including data loss, parallax errors, the inconvenience and distracting effect of wearables, and/or the need for several cameras to capture each person. Here we present a novel eye-tracking solution, consisting of a dual-camera system used in conjunction with an individually optimized deep learning approach that aims to overcome some of these limitations. Our data show that this system can accurately classify gaze location within different areas of the face of two interlocutors, and capture subtle differences in interpersonal gaze synchrony between two individuals during a (semi-)naturalistic face-to-face interaction.

**Supplementary Information:**

The online version contains supplementary material available at 10.3758/s13414-023-02679-4.

## Introduction

Synchronization and coordination of gaze during face-to-face human interaction modulate visual attention, and provide information about the subtle aspects of the interaction, such as the individual’s intentions, the presence of mutual gaze, and the affective context within which the interaction takes place (Bayliss, Schuch, & Tipper, 2010; Behrens et al., 2020; Brône Geert, 2018; Hari, Henriksson, Malinen, & Parkkonen, 2015; Kragness & Cirelli, 2021; Wohltjen & Wheatley, 2021). To objectively investigate these processes is not an easy undertaking, yet it is motivated not only from the perspective of basic research, but also for clinical applications. Indeed, deficits in non-verbal communication are defining features or common characteristics of a wide range of clinical conditions, including e.g., various neurodevelopmental disorders, traumatic brain injury, and dementia (American Psychiatric Association, 2013; Banovic, Zunic, & Sinanovic, 2018; Goldberg et al., 2021; MacDonald, 2017; Marrus & Hall, 2017; Missiuna & Campbell, 2014).

Important insights regarding social attention have been gained by showing (non-interactive) pictures or movies of human faces on a monitor, while simultaneously tracking the gaze patterns of an observer. Such setups have been used to show, for instance, how gaze is distributed between the upper (eyes) and the lower parts (mouth) of a face during different tasks and conditions (Laidlaw, Risko, & Kingstone, 2012; Lansing & McConkie, 1999, 2003; Lusk & Mitchel, 2016), how gaze distribution changes across development (Irwin, Brancazio, & Volpe, 2017), and how it differs between groups, including in typically developing individuals and those diagnosed with developmental disorders such as autism (Pelphrey et al., 2002; Senju & Johnson, 2009). For instance, these studies have demonstrated that as typical infants acquire language, their gaze becomes more focused on the lower parts of the face (Tenenbaum et al., 2015), which begins at approximately 6 months of age (Hillairet de Boisferon, Tift, Minar, & Lewkowicz, 2018; Lewkowicz & Tift, 2012; Pons, Bosch, & Lewkowicz, 2015). They have also shown that individuals diagnosed with autism often show reduced amount of gaze directed to the eyes (Chita-Tegmark, 2016). Finally, such setups have revealed the presence of a left visual bias, or preferential looking to the left half of another person’s face (from the viewer’s perspective), e.g., Butler et al., 2005; Guo, Meints, Hall, Hall, & Mills, 2009, and have found this bias to be less distinct in individuals with autism, dyslexia, or depression (Dundas, Best, Minshew, & Strauss, 2012; Guillon et al., 2014; Masulli, Galazka, Eberhard, & Johnels, n.d,; Åsberg Johnels, Galazka, Sundqvist, & Hadjikhani, 2022).

However, while research on gaze behavior to non-interactive stimuli presented on a monitor screen has informed much of the basic and applied research, visual attention might operate differently in face-to-face interactions, when two people are looking, and influencing each other’s behavior (Risko, Laidlaw, Freeth, Foulsham, & Kingstone, 2012). A few creative attempts have been made to try to capture the gaze of both partners simultaneously in face-to-face interaction, for instance by using eye tracking glasses (Behrens et al., 2020; Broz, Lehmann, Nehaniv, & Dautenhahn, 2012; Cañigueral, Ward, & Hamilton, 2021; Franchak, Kretch, & Adolph, 2018; Ho, Foulsham, & Kingstone, 2015; MacDonald, 2017; Prochazkova, Sjak-Shie, Behrens, Lindh, & Kret, 2022; Rogers, Speelman, Guidetti, & Longmuir, 2018; Yu & Smith, 2013, 2017)or by having participants view their partner live on a screen while their gaze patterns are recorded, much like during a typical video call (e.g., Skype or ZOOM) (Hessels, Holleman, Cornelissen, Hooge, & Kemner, 2018; Holleman, Hessels, Kemner, & Hooge, 2020; Holleman et al., 2021). A number of practical and technical issues still remain problematic with these approaches.

First, in regard to eye tracking glasses, these have been shown to be less tolerated by individuals with sensory issues, and/or difficulties staying still, such as those on the autism spectrum and/or with attention-deficit hyperactivity disorder (Alcañiz et al., 2022; Cascio et al., 2008; Kyriacou, Forrester-Jones, & Triantafyllopoulou, 2021; Niehorster et al., 2020). Evidently, eye tracking glasses can be cumbersome, distracting, and hindering natural interactions more generally. Indeed, in the study by Wohltjen and Wheatley (2021), the instructions given to the participants encouraged them to ignore the glasses “that look[ed] a little funny” and to remain still, “because the glasses don’t work as well when you move around” (p. 7). Moreover, while the manufacturers of eye tracking glasses^1^ report a maximum accuracy varying between 0.3 — 1.0 degrees (Cognolato, Atzori, & Müller, 2018; Kassner, Patera, & Bulling, 2014), in a comprehensive experimental evaluation, the actual accuracy was often considerably lower^2^ (between 0.8 — 3.7 degrees in the calibrated plane and with an additional 0.8 — 3.1 degrees during facial movement; Niehorster et al., 2020). In consequence, studies of face-to-face gaze using eye tracking glasses have not been able to provide data with sufficient quality to allow a fine-grained gaze analysis of areas of interest within the observed face, which is needed to distinguish gaze between upper or lower parts, left or right side, or to assess gaze synchronicity such as during mutual eye contact (Behrens et al., 2020; Prochazkova et al., 2022).

Second, using live displays of the participants on screens addresses limitations regarding accuracy and the possible discomfort of using wearables. However, at the same time, this approach introduces other restrictions when it comes to the naturalness (Valtakari et al., 2021) and the perspective dynamics of the interaction (Tran, Sen, Haut, Ali, & Hoque, 2020). In regard to the latter, in cases when the camera has a different perspective than the participant’s sight, a so-called eye-contact parallax error is created, which hinders natural face-to-face synchrony (Tran et al., 2020). The eye-contact parallax error is evident for us in video calls when we often are not sure if our conversational partner is looking into our eyes. In one recent attempt to address this, Holleman et al., (2021) (see also, Hessels et al., (2018) and Holleman et al., (2020)) used half-silver mirrors to record the participants' faces from behind the preview, which enabled a closer approximation of natural interaction. Still, as highlighted by Valtakari et al., (2021): “not using screens […] is arguably often more representative of a typical face-to-face conversation that a person might have on a regular day” (p. 1600). Thus, developing simultaneous gaze tracking solutions that are not dependent on either wearables or screens (2D surface on which a face is projected) is extremely worthwhile.

A third potential approach worth noting, that does not require the use of monitors or wearables, is often referred to as scene-based approach. In these types of setups, eye tracker(s) have been placed on the table, and other camera(s) are placed closely above the head (Vehlen, Spenthof, Tönsing, Heinrichs, & Domes, 2021) or between interacting participants (Falck-Ytter, 2015) in order to track gaze of one person to another. Although less intrusive than the wearables and more direct than monitors, this approach has not, to our knowledge, been used to examine the gaze of both interlocutors. Extending this less intrusive approach to simultaneous eye tracking within a 3D coordinate system would require to account for the locations and orientations of the participants’ eyes to obtain stable gaze estimates. As of yet, this has not been done for scene-based approaches using commercial eye trackers.

**Fig. 1:**
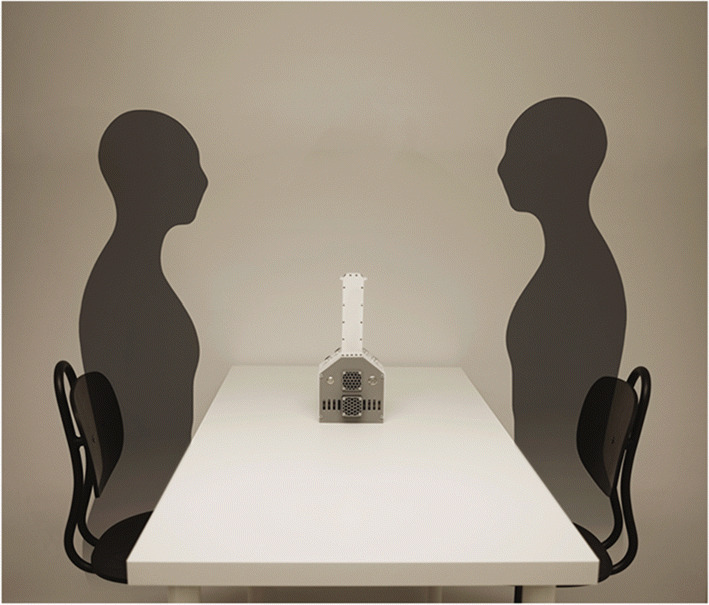
i+i, an end-to-end dual-camera system for face-to-face interaction. The cameras are located in the center between participants at the height of 29.5 cm from the table (at the top of the casing, positioned opposed to the other) and directed at an angle of 30 degrees from the horizontal plane. The participants are sitting so that their eyes are equally high, at approx. 60 cm above the table, by adjusting the chairs. The device pillar is 3 cm wide, and the bottom box encloses the full recording hardware (Jetson Nano)

Building on previous efforts, we have developed a novel dual-camera system, dubbed i+i, that uses deep learning-based eye tracking to better account for the location of the eyes and the orientation of the gaze for both participants in a shared 3D coordinate system (Gibaldi, DuTell, & Banks, 2021). At a typical interpersonal distance of 60 cm, a 2-degree angle accuracy is needed to categorically discriminate gazed-at areas of the face (left eye vs. right eye vs. the mouth). Thus, 2-degree accuracy was considered a target value in the present proof-of-concept study. In addition to the discrimination of facial features, we wanted to explore if the data were sufficiently accurate for identifying gaze movement synchronization between the two interacting individuals. Along with developing dedicated software, we designed a compact setup that is no bigger than a vase, and that is meant to not be overly distracting during the face-to-face interaction (see Fig. 1).

## Method

### Participants

An interacting dyad consisted of an experimenter (26-year-old male) and a participant. A convenience sample of 8 healthy participants (4 women) with a mean age of 32.5 years (range: 23 – 44) was recruited as interacting dyads.

### Data preparation and neural network optimization

In order to create a compact, single capturing device, a Jetson Nano, Developer Kit, was used with dual Raspberry Pi camera modules v2 (Sony IMX219 8-megapixel CMOS image sensors, 1/4” format, focal length = 3.04 mm, aperture = 2.0) mounted within a custom 3D-printed casing. The frames from the two cameras were synchronized at the software level, based on the timestamps that were recorded for each frame, taking advantage of Nvidia’s hardware-accelerated video encoding with minimal lag. Head width and location of the left eye, right eye, mouth, and nose were measured with a digital vernier caliper to guarantee accurate pose and distance estimation. Bounding boxes, enclosing the manually measured facial landmarks, were labeled on videos previously recorded by the camera system using the graphical image labeling tool LabelImg (Tzutalin, 2015) and iris segmentation maps, using custom-made, OpenCV-based software for semantic segmentation (Bradski, 2000). The neural network training was conducted through repeated K-fold cross-validation (except for YOLOv4, which has its framework-specific training) on the frames from all individuals’ first two videos to output the image position of the eye rotation center, which was estimated by using the inverse rendering of the eye model, directed toward calibration stimulus. Repeated K-fold cross-validation was chosen due to its known efficacy in using a low amount of data while allowing low model bias and high accuracy (Kuhn & Johnson, 2013). Kalman filtering was applied to all neural network outputs to make facial landmarks and pose estimations closer to reality (Diaz Barros, Mirbach, Garcia, Varanasi, & Stricker, 2019). The bounding boxes were detected using YOLOv4 (Bochkovskiy, Wang, & Liao, 2020), and enhanced by a convolutional autoencoder for center estimation, an approach that has shown excellent accuracy for pupil segmentation (Zdarsky, Treue, & Esghaei, 2021).

The eyeball and iris are often represented as larger and smaller spheres to estimate the gaze (Park, Zhang, Bulling, & Hilliges, 2018; Sun, Liu, & Sun, 2015; Swirski & Dodgson, 2013; Wood et al., 2015; Yiu et al., 2019). By using the inverse rendering of the projections of the iris and eyeball on the image plane, the 3D location and orientation can be estimated (Safaee-Rad, Tchoukanov, Smith, & Benhabib, 1992). In other words, the projected coordinates on the image plane representing the eye and iris center can provide an estimation of gaze direction (e.g., Tsukada, Shino, Devyver, & Kanade, 2011). In order to provide stability in the estimation of gaze estimation during head movement, we used the eye model as implemented by Tsukada et al., (2011) in combination with an adaptation of the elliptical representation of the iris (Swirski and Dodgson, 2013), by identifying the eye for each frame (e.g., Dierkes, Kassner, & Bulling, 2019). Further, we accounted for the head pose and location based on the facial landmarks which have been shown to be useful for gaze estimation in extreme poses (Valenti, Sebe, & Gevers, 2012).

Based on the output, a plane centered between the eyes and the mouth was estimated, and the intersection point of the second individual’s gaze to the first individual’s facial plane was calculated (see Appendix A: 2.1 Estimation of face plane). Gaze was estimated using an adaptation, of the 3D eye model presented by Swirski and Dodgson (2013), which intended to allow more head movement. The model continuously used the distance between the estimated image position of the eye and pupil center to obtain the gaze vector while accounting for the estimated head pose and location. Closed and open eyes were manually labeled on images obtained from the iris bounding boxes, obtained from the YOLOv4 detection, to train a binary convolutional neural network classifier. No manual labeling was performed on the videos that were used for evaluating performance. When gaze from a specific camera was analyzed, the filtered time series from the opposed camera were linearly interpolated to the timestamps of the specific camera. The pre-processing of the data and the training and application of the neural networks were done offline.

### Precautions for application in a face-to-face setting

Several precautions were made to ensure a controlled experimental setting that would be suitable for both gaze-to-screen and face-to-face interaction. First, the lighting conditions were controlled, to create a diffuse, non-distracting light (approx. 1000 lux, similar to home studio lighting), while at the same time obtaining favorable conditions for the video recording. Lighting conditions were kept constant during both experiments. Second, each camera was calibrated with a rectangular grid as implemented in the OpenCV library (Bradski, 2000), and the side of the camera system, where the experimenter and participant sat, was rotated between sessions. This was done in order to minimize the effect of possible camera differences. Finally, precautions were made to make the training data generalizable to the face-to-face setting. The monitor was set up with its center at the level with the participants’ eyes. A second condition, in which the participant was instructed to move his/her head while looking at the calibration stimulus, was added to get more variance in the participants’ poses. Data augmentation such as image rotation, translation, and scaling were also added to increase the variability of the data. These data augmentation techniques are part of the default implementation of YOLOv4 (Bochkovskiy et al., 2020) and have previously been successfully implemented for pupil segmentation, using a U-Net-based architecture (Yiu et al., 2019).

### Experiment 1

Gaze angle accuracy was assessed in eight participants and the experimenter. Three conditions of a smoothly moving nine-point calibration were presented on a monitor, during which participants were asked to follow these points while [1] sitting naturally and comfortably without excessive movement, [2] performing spontaneous head movement, and [3] doing the same as in [1]. Data from the first two presentations (Conditions 1 and 2) were used to optimize the weights of a convolutional neural network, and the third presentation was used for unbiased evaluation of the generalization error of the final model (equivalent to a test dataset).

The 2D coordinates of the intersection between the gaze vector and the screen were estimated using the line plane intersection equation (see Appendix A: 2.2 Line plane intersection). The coordinates were then averaged across the left and right eyes. To calculate the angular gaze metrics (which are needed to evaluate the quality of the gaze estimation), the Euclidean distance between the estimated and expected gaze coordinates was converted to degrees, by taking the inverse tangent of the distance to the target point, divided by the distance to the participant or experimenter.

### Experiment 2

Coordination of gaze between individuals in the dyad was examined here. The same eight participants sat in front of the experimenter with the camera system placed between them on a table (see Fig. 1), and each dyad followed pre-recorded verbal instructions of where to look – left eye, right eye, mouth, or just outside the face^3^ – presented in pseudo-random order across 60 5-second trials. Both the participant and experimenter were instructed to look at one explicit facial area of the person in front of them until a new area was named while maintaining their position, as they would during a natural conversation. We did not control that the participants looked at the specific area, but had no reason to believe that they would not do so.

Since both the participant and the experimenter followed the same instructions, we prompted synchronization in the form of looking at similar areas. This specifically involved mutual eye contact when the participant and the experimenter were prompted to look at the left and right eye conditions. This paradigm also intended to evaluate the synchronization of gaze rotation, since both individuals shifted their gaze from one area to another nearly simultaneously after a prompt.

The median gaze intersection points of the face plane of the opposed individual, acquired from the first trials of the left- and right eye and the mouth conditions, were used to form the matrix for affine transformation, which was applied to the intersection points of the face plane (see Appendix A: 2.3 Calibration of face plane); these trials were excluded from the statistical analysis. To create moving areas of interest (AOI), we followed the procedure for automatic AOIs described by Hessels, Benjamins, Cornelissen, and Hooge (2018), which we adapted for our specific areas. Gaze points at the face plane located more than approximately 3 degrees (6.3 cm) away from any of the facial landmarks were classified as outside of the face. If the distance was smaller, the label of the closest landmark was selected (see Appendix A: 2.4 Classification of gaze on facial areas).

## Results

### Experiment 1

The video that was not used in neural network training served for the unbiased evaluation of the final model. Data points were classified as eyeblinks or outliers, obtained by a likelihood threshold in line with the procedures of deep learning eye tracking (Zdarsky et al., 2021), together represented 7.95% of the data.

Median gaze angle resolution was 2.04 degrees (range 0.98 – 2.74, *SD* = 0.70, *n* = 9 [including experimenter]), (2.56 cm), which, assuming a distance of 60 – 80 cm, is within the estimated range necessary to provide the distinction of facial gaze areas in face-to-face interaction.

**Fig. 2:**
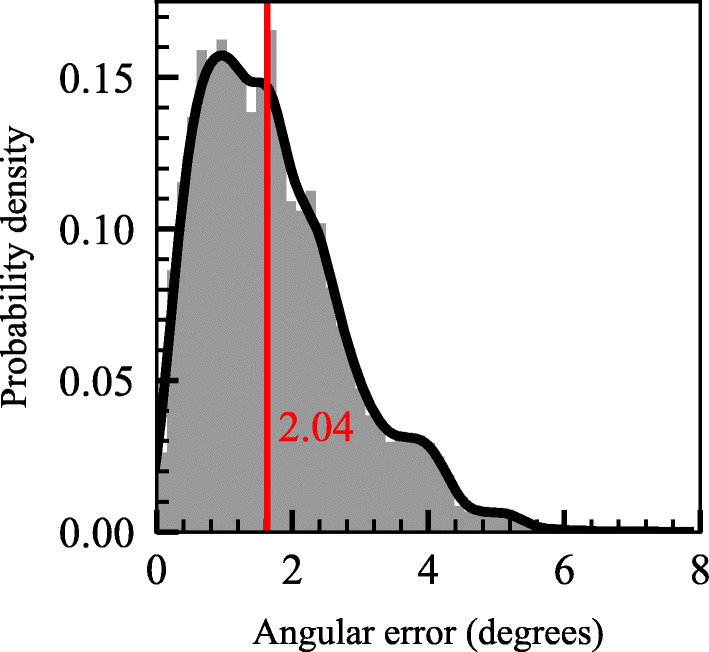
Gaze angle accuracy during novel nine-point calibration stimulus. Kernel density estimation from all included data points (*n* = 9740), and the median across all participants and the experimenter (based on median per individual, *n* = 9), is displayed in red

The experimenter only performed the calibration conditions once and had a median accuracy of 2.74 degrees (2.84 cm), i.e., roughly similar to the participants’ median. The median gaze error was estimated from the captured data collected during the condition in which the participant or the experimenter was expected to look at each of the nine calibration points. The kernel density estimation of gaze angle error is displayed in Fig. 2. Additional information and metrics related to the performance of the gaze estimation, such as robustness, accuracy, and precision for Experiment 1 are located in Appendix B: Table 1.

### Experiment 2

Statistical analyses were based on the last 4 seconds of each trial (which corresponded with the time frame starting after completion of verbal instructions, that lasted between 0.4 to 0.6 seconds).

In the second experiment, an average of 12% of the data were excluded per participant. No participant or trial was excluded, and the eye blink classifier identified 9.03% of the excluded data as eyeblinks. Yet, this needs to be interpreted with caution since a binary image classifier like ours risks classifying extreme poses as eyeblinks if neither the iris nor the sclera is visible.

**Fig. 3:**
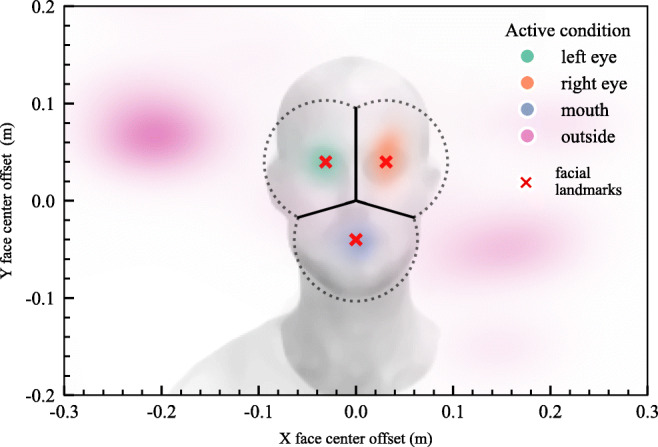
Kernel density estimation of the participants’ gaze on a demonstrative image (*n* = 8); colored by active condition. Gaze was classified according to the areas: upper left face area = *left eye*, upper right face area = *right eye*, lower face area = *mouth*, and outside the dotted line = *outside*

**Fig. 4:**
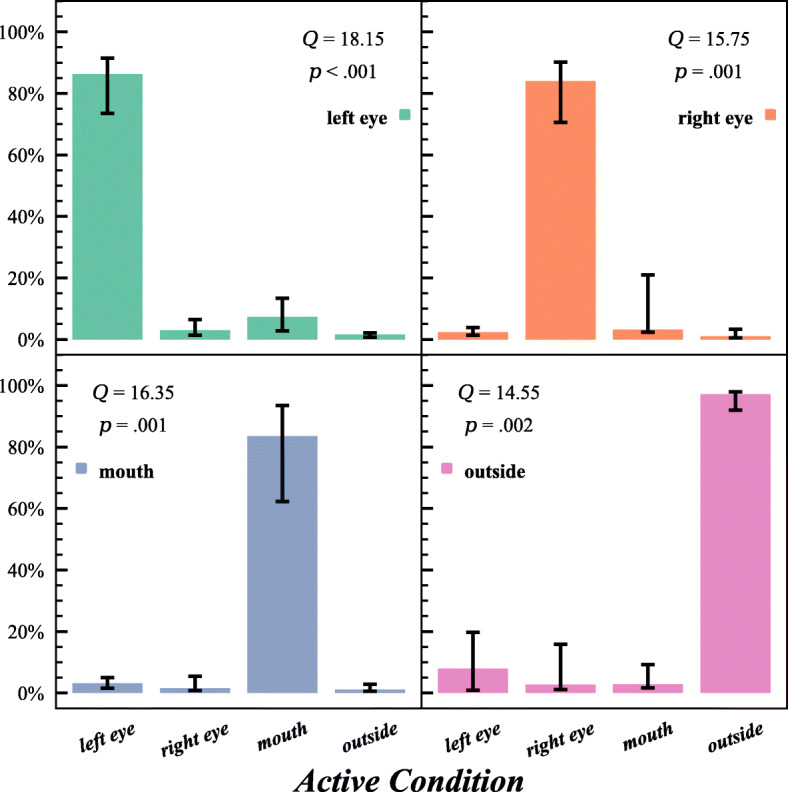
Median prediction rate in specific conditions, compared with prediction rate in other conditions. *Q*-statistics and *p*-values from Friedman’s test in the upper corners. Error bars represent upper and lower quartiles (*n* = 8)

A 2D kernel density estimation was made for the points of intersection on the facial plane to display the closeness to the generated AOIs (see Fig. 3). The median rate of correct gaze classification was 89.54% across participants and conditions. Four separate Friedman tests confirmed that there were significant differences between the median prediction rates in each of the conditions *Q* (*df* = 3, *n* = 8) ≥ 14.55, *p*≤ .002 (see Fig. 4 illustrating the expected patterns). Reciprocally, the median rate of correct classification of the experimenter’s gaze to the participants’ faces was 93.60% (*p* ≤ .002).

Given that the rates of gaze data for the expected areas were considerably higher than for the other conditions, these findings show that the system is capable of providing high accuracy for the discrimination of gaze to different facial areas in the face-to-face interaction, such as whether the observer looked at the face vs. next to it, whether the observer looked at the eyes or the mouth, and even more specifically which eye was looked at. Median gaze was here estimated following the steps presented in Experiment 1, but now by using the intersection of the observer’s gaze to the plane of the experimenter’s or the participant’s face, instead of the plane of the monitor. Median gaze angle resolution, based on the 4 last seconds of each trial, was here 2.51 degrees (2.74 cm) for the eight participants and 2.48 degrees (2.84 cm) for the experimenter looking at the participants. Additional metrics related to robustness, accuracy, and precision for Experiment 2 are located in Appendix B: Tables 2 and 3.

We finally evaluated mutual coordination of gaze (interpersonal synchrony) by comparing data from the first 2 seconds, of the synchronized vs. not synchronized sequences of gaze angles (including both eye and head movement), using time-lagged cross-correlation (see Appendix A: 1. Movement synchronization analysis). Gaze angles were chosen instead of position or categorical facial area, because we wanted to investigate the synchronization of coordinated gaze between the individuals, rather than similarities in where they looked. Unsynchronized data consisted of data segments separated by 5 seconds, to keep the external properties as constant as possible. Correlations and temporal offsets (the time lag where the maximum correlation was identified) were condensed as median per participant, to be suitable for individual-based statistics.

The synchronized sequences had a higher median correlation (0.60 vs. 0.47) and a lower median time lag at time points where the maximum correlation between the sequences occurred (0.30 vs. 0.54 seconds); Wilcoxon signed-rank tests revealed significant differences in both the temporal offset (*n* = 8, *W* = 1.0, *p* = .017) and in the correlation between the synchronized and unsynchronized sequences (*W* = 0.0, *p* = .012). This implies that our system allows estimating 3D gaze angles that are precise enough to differentiate changes in interpersonal gaze synchronization during face-to-face interaction.

## Discussion

Eye gaze research has revealed critical findings that have helped our basic understanding of social processes, and of their limitations in individuals who have difficulties in communication, such as those with neurodevelopmental disorders. Setups that simultaneously study the gaze of two individuals – in face-to-face interactions – have used several strategies, each with their own drawbacks.

Our results demonstrate that i+i, our novel dual-camera system, can estimate gaze to an angular accuracy of approximately 2 degrees from both partners in face-to-face interaction while addressing some of the drawbacks of previous solutions. This is comparable with current deep learning eye tracking using one camera (Rakhmatulin & Duchowski, 2020; Zdarsky et al., 2021), and seems sufficient to accurately differentiate gaze directed towards different parts of the face of each interlocutor during face-to-face interaction. In addition, our system shows to be accurate enough for identifying gaze movement synchronization during dyadic interaction. In sum, i+i permits the direct recording of participants’ gazes without the use of wearable devices or monitors to display the participants, thereby allowing face-to-face interaction to occur more naturalistically and without obvious distractions.

Some comparisons to other solutions can be highlighted. First, regarding eye-tracking accuracy, a recent study (Vehlen et al., 2021), utilized a Tobii X3-120 (Tobii, 2011) eye tracker that was combined with camera(s) above the head, and tested in a face-to-face situation with one participant gazing at an experimenter. That study obtained an impressive accuracy of 0.7 degrees during the conversation (Vehlen et al., 2021), i.e., superior to ours, and not far from the manufacturer-reported accuracy of 0.4 degrees with gaze-to-screen, without chinrest. However, whether non-wearable commercial eye trackers with compatible software can be used to accurately explore simultaneous gaze of two partners in a 3D coordinate system is still to be shown empirically. It is important to note that the i+i was specifically developed for this purpose, and has not been tested in a context that would ensure maximal performance accuracy (e.g., with people being instructed to be fully stationary, or by using a chinrest). Nonetheless, the possible parallax error from estimating gaze to a stationary plane, present in the scene-based setups (which is different from the eye-contact parallax) needs to be considered, which the i+i addresses through a 3D coordinate system (Gibaldi et al., 2021). Moreover, recent studies have shown limitations of commercial eye tracking glasses in providing discrimination of different facial features (Behrens et al., 2020; Prochazkova et al., 2022). Also, some individuals with sensory and neurodevelopmental issues may experience discomfort with wearables (cf. Alcañiz et al., 2022). Our system may therefore also provide a non-wearable alternative to the commercial wearable eye tracker. It is, however, important to know that while the hardware used for i+i is readily available, yet, as of now, the handling requires programming knowledge. With regards to live monitor displays, the question of the best choice of setup depends on the requirements of the research question. If the naturalness of the face-to-face interaction is important, the i+i might be preferable. On the other hand, if accuracy is of main importance, then displays on a monitor might be preferable (Hessels et al.,, 2018; Holleman et al.,, 2020, 2021).

Our study further underscores the usefulness of neural networks in the analysis of gaze patterns (Ba & Odobez, 2006; Capozzi et al., 2019; Kellnhofer, Recasens, Stent, Matusik, & Torralba, 2019; Massé, Ba, & Horaud, 2018; Otsuka, Yamato, Takemae, & Murase, 2006). Such approaches have previously been used for the automatic quantification of head orientation and visual attention (Ba & Odobez, 2006; Capozzi et al., 2019; Massé et al., 2018; Otsuka et al., 2006). For example, Kellnhofer et al., (2019) introduced an interesting 360-degree panoramic camera solution using a long term-short memory (LTSM) network for estimating the gaze of multiple people with 8-degree accuracy. Thus, when further refining the accuracy of a non-wearable, monitor-less simultaneous eye tracking, neural network approaches are likely key.

A couple of methodological aspects regarding our deep learning approach are important to highlight. First, it allowed to provide stable predictions in a face-to-face setting. However, it is important to note that the neural networks were trained on data acquired from gaze-to-a-monitor, and that steps were taken to increase the generalizability to face-to-face interaction. An affine transformation was used to calibrate the predictions to the new face-to-face setting. Although we utilized three calibration points in the face (right eye, left eye, and mouth), we believe that using (at least) one additional facial calibration point may in the future further improve the accuracy of the transformation matrix (without adding unnecessary complexity, cf. Lara-Alvarez and Gonzalez-Herrera, 2020). Second, an improvement consist in training the models on actual face-to-face gaze data, which would eliminate the need for a monitor during calibration. While such context-specific training may reduce the need for a subject-specific training, it may need to be based on a larger amount of data, due to increased noise.

Aside from methodological aspects of neural network training, limitations specific to the present study should be mentioned. First, our results are based on a limited sample size. Although this is comparable to other studies using neural network-based approaches (Yiu et al., 2019; Zdarsky et al., 2021), it is smaller than a similar study analyzing face-to-face interaction (Vehlen et al., 2021). Second, 12% of the data were excluded in the face-to-face experiment (Experiment 2) but it is comparable to the approx. 11% reported by Vehlen et al., (2021). Third, we did not specifically analyze robustness to head movement, although we included data with spontaneous head movement from the participants when training the neural networks (in Experiment 1, Condition 2), which very likely provided more stability (cf. Zdarsky et al., 2021).

In conclusion, our dual-camera system was specifically developed and evaluated for its usefulness as a tool in research on gaze patterns and synchronization in face-to-face interactions without the use of previewing monitors or wearables. The results from the present proof-of-concept study show that i+i is potentially useful for this purpose.

## Electronic supplementary material

Below is the link to the electronic supplementary material.

(PDF 216 KB)

## Electronic supplementary material

Below is the link to the electronic supplementary material.

(PDF 281 KB)

## Data Availability

Neither of the studies reported in this article were preregistered. The data have not been made available on a permanent third-party archive because participants were not asked to consent for their data to be made publicly available, even when anonymized. Data are available upon request from those who wish to collaborate with us, via a Visitor Agreement with the University of Gothenburg, if appropriate, and under the existing ethical approval. In detail steps of the analysis procedures are available in the Supplemental Material associated with this article. Representative scripts used to analyze the data are posted publicly and accessible here: https://github.com/thoraxmax/face-to-face-interaction-analysis.

## Footnotes

1 of the brands and models **Pupil labs:** Pupil Pro headset; **Arrington Research:** BSU07— 90/220/400, Ergoneers Dikablis, ISCAN OmniView; **SMI:** Eye Tracking Glasses 2 Wireless; **SR research:** EyeLink II with Scene Camera; **Tobii:** Tobii Pro Glasses 2

2 of the brands and models **Tobii:** Tobii Pro Glasses 2; **Grip:** Pupil headset; **Pupil labs:** Pupil headset; **SMI**: Eye Tracking Glasses 2 Wireless

3 Translated directly from the Swedish word ***bredvid*** the preposition implies, in this specific context, that the participant will look somewhere within the vicinity of the face, but not the face directly.
