# Supplementary material for: A novel end-to-end dual-camera system for eye gaze synchrony assessment in face-to-face interaction
Source: Atten Percept Psychophys. 2023 Apr 26;86(7):2221–30. doi: 10.3758/s13414-023-02679-4 (PMC11480169; doi:10.3758/s13414-023-02679-4)
Supplement: Supplementary file 1 — (PDF 216 KB) [file 13414_2023_2679_MOESM1_ESM.pdf]

Appendix A

M. Thorsson et al.

The supplementary text serves as an extended method to the paper, *A novel end-to-end dual-camera system for eye gaze synchrony assessment in face-to-face interaction.* Mathematical formulas and Python 3.8.5 functions, used in our methodology are presented below.

1 Movement synchronization analysis

Time-lagged cross-correlation (TLCC) is a frequently used method for evaluating relationships between time series. In recent years, this method has been utilized for evaluating movement synchronization (Amblard, Assaiante, Lekhel, and Marchand, 1994; Behrens, Moulder, Boker, and Kret, 2020; Cornejo et al., 2018; Derrick, 2004; Ramseyer and Tschacher, 2008; Reidsma, Nijholt, Tschacher, and Ramseyer, 2010). Here we extend the use of time-lagged cross-correlation method to evaluate the synchronization of gaze angles. We do so by first explaining its use in one-dimensional data in order to clarify our actual 3D implementation.

1.1 Cross-correlation

The cross-correlation can be used to estimate the similarity between two numerical arrays, as in our case, time series sequences from individual **A** and individual **B**. Let σ denote the standard deviation of the time series. The cross-

correlation, r, is the normalized cross-covariance between two time series, **A** and **B** given by the equation

$$r = \frac{\mathrm{cov}(A, B)}{\sigma_A \sigma_B}$$

where

$$\mathrm{cov}(A, B) = \frac{\sum(A - \bar{A})(B - \bar{B})}{n - 1}.$$

1.2 Time-lagged cross-correlation

The cross-correlation can be lagged in discrete steps to identify the step with the highest correlation. In our face-to-face experiment, a relationship between the individuals' gazes is expected, due to being instructed where to look (for example from looking at each other's mouth, to looking at each other's left eye). Naturally, it is not certain that the reactions occur at the exact same time or that the eye movements have identical angular patterns. The time-lagged cross-correlation is therefore used to determine the amount of time (time lag), between the two time series where the maximum correlation is located. The time of 2.0 seconds was chosen to account for the duration of the verbal instruction (between 0.5 – 0.7 seconds) and in order to include the full key eye movement including (A) *the stationary time period before initiation*, (B) *the movement execution* and (C) *the stationary time period after execution.* This is done to maximize the chance for the cross-correlation to be estimated at the lag where the key movements are matched between individuals. We chose a maximum lag of 1.0 seconds, to allow us to analyze a minimum of 60 data points (the camera's frame rate is 60 Hz), which is in accordance with the guidelines presented and used in current research (Behrens et al., 2020; Schoeneberger, 2016). The below function, *lagged_cross_corr(A, B, lag)*, is used to estimate the correlation coefficient, r, for the specific lag:

```python
def lagged_cross_corr(A, B, lag):
  if lag > 0:
    r = np.corrcoef(A[lag:], B[:2 * fps - lag])[0, 1]
  else:
    r = np.corrcoef(B[-lag:], A[:2 * fps + lag])[0, 1]
  return r
```

An executable Python example, *TLCC.ipynb*, is accessible at the Github repository (Thorsson, 2022):

github.com/thoraxmax/face-to-face-interaction-analysis/blob/main/TLCC.ipynb

1.3 Comparing synchronized to unsynchronized sequences

There is an increased probability to make false conclusions based on the analysis of synchronized sequences alone, due to the risk of random fluctuations or other mutual outer factors such as changes in lighting conditions. Therefore, we compare *synchronized* to *unsynchronized* time series sequences (Ramseyer and Tschacher, 2008). In order to keep external factors constant, the unsynchronized sequences consist of the first individual's gaze angles, matched to sequences 5.0 seconds further in time of the second individual.

1.4 3D implementation of lagged cross-correlation for Euler angles

The gaze unit vector $\hat{OG}$, starts at the center of the eyes, $\underline{o}$, and ends at the point of gaze, $\underline{g}$, which is given by the equation

$$\hat{OG} = \frac{\vec{OG}}{|\vec{OG}|}$$

where

$$\vec{OG} = \underline{g} - \underline{o}.$$

Let $\hat{Z}$ denote the unit vector, as the negative Z-axis,

$$\hat{Z} = \begin{bmatrix} 0 \\ 0 \\ -1 \end{bmatrix}.$$

Finally, we estimate Euler angles based on the function, from the SciPy library (Virtanen et al., 2020):

```
from scipy.spatial.transform import Rotation as R
euler_angles=R.align_vectors(OG, Z)[0].as_euler('xyz', degrees=False)
```

Euler angles are known to be intuitive but also carry some mathematical inconveniences related to gimbal lock and angular periodicity. Due to the advantage of our camera system to record the participants from the front, we will not need to address the problem with gimbal lock. In order to address the problem of angles being periodic, we first transform each time series by iterating the below function:

```
def fix_degrees(a):
  return np.cumsum([normalize_angle(i) for i in np.diff(a)])
```

The function is used to first estimate the difference between the angles, followed by applying a corrective function, *normalize_angle(x)*, and calculate the cumulative sum using the now adjusted angular delta.

The function to normalize the angle is based on the implementation used in R. Labbe's Kalman filter package, FilterPy (Labbe, 2014):

```
def normalize_angle(x):
  x = x % (2 * np.pi)
  if x > np.pi:
    x -= 2 * np.pi
  return x
```

The presented method, to adjust the angular data and calculate the cross-correlation, is applied to the three Euler angles. The average correlation, $\bar{r}$, is based on cross-correlation independently for the three angles (x, y, and z) between the two individuals' time series sequences. The average correlation is given by the equation

$$\bar{r} = \frac{\text{cov}(A_x, B_x) + \text{cov}(A_y, B_y) + \text{cov}(A_z, B_z)}{\sigma_{A_x}\sigma_{B_x} + \sigma_{A_y}\sigma_{B_y} + \sigma_{A_z}\sigma_{B_z}}$$

and is estimated for each lag. The lag with the highest correlation is located and used as an absolute value in our statistical analysis.

Our experiment is structured by timed events with verbal instructions. These events are used as logical divisions of the data, an approach used in other time-structured analyses of coordinated movement (Amblard et al., 1994; Cornejo et al., 2018) instead of analyzing the full time series, or through rolling windows, which are solutions that have been used in more unconstrained interaction experiments (Behrens et al., 2020).

2 Gaze in face-to-face interaction

2.1 Estimation of face plane

To ease the estimation of facial gaze location, a plane is used to represent the facial surface. This requires the 3D location and orientation of the head.

Five facial landmarks are used for the pose estimation to form a matrix of known locations. The 3D location of the left eye, right eye, and the position between the nostrils, and the upper lip, are manually measured.

The 2D image position is detected by using a custom-trained YOLOv4 network (Bochkovskiy, Wang, and Liao, 2020) for detecting bounding boxes containing the corresponding landmarks.

3D translation and rotation are estimated by setting up a *Perspective-n-Point problem*, which can be solved through minimization. An example of this

is the function,

cv2.solvePnP(objectPoints, imagePoints, cameraMatrix, distCoeffs) as implemented in the OpenCV computer vision library (Bradski, 2000).

After pose estimation, we estimate the average location between the left and right eye and the mouth. The pose is estimated based on the measurements of the individuals which were centered at the nose. We assume face symmetry for simplification. The matrix, M, which was used as *objectPoints*, includes the known locations of the landmarks and is given by

$$M = \begin{bmatrix} eye_y & eye_x & -eye_z \\ eye_y & -eye_x & -eye_z \\ 0 & 0 & 0 \\ -mouth_y & 0 & mouth_z \end{bmatrix}.$$

2.2 Line-plane intersection

To estimate the point of intersection of the face plane (or another plane such as a monitor) and the other individual's gaze, a *line-plane intersection* equation is used. All orientations are transformed into unit vectors, and the translation vectors are used in the computations. The below function is used to estimate the point of intersection:

```
def LinePlaneCollision(planeNormal, planePoint, rayDirection, rayPoint):
    ndotu=planeNormal.dot(rayDirection)
    w = rayPoint - planePoint
    si = -planeNormal.dot(w) / ndotu
    Psi = w + si * rayDirection + planePoint
    return Psi
```

The function is based on the terminology from Practical Geometry Algorithms (Sunday, 2021) and the implementation by Sheerman-Chase (2016).

2.3 Calibration of face plane

In our implementation, the neural networks were trained on a screen stimulus, which motivates the need for a subject-specific calibration, known to be required for optimal accuracy (Schaller et al., 2021). We, therefore, performed a simple correction using the first occurrence of when the individual looks at the left eye, the right eye, and the mouth. The median of each intersection point distribution is used to form the affine transformation matrix, which maps the point to the corresponding known facial landmark. The matrix is then applied to correct the following intersection points.

The affine transformation matrix is estimated using the function, *cv2.estimateAffine2D(src,dst)*, from the OpenCV computer vision library (Bradski, 2000).

An executable Python example, *face_plane.ipynb*, is accessible at the Github repository (Thorsson, 2022):

github.com/thoraxmax/face-to-face-interaction-analysis/blob/main/face_plane.ipynb

2.4 Classification of gaze on facial areas

The facial areas are generated based on the automatic generation of areas of interests presented in current research (Hessels, Benjamins, Cornelissen, and Hooge, 2018) adjusted for three facial areas of interest. A radius of approximately 3 degrees (6.3 cm) is used as the minimum distance to classify a point of intersection as outside the face. The points within this threshold are classified as their closest neighbour.

First, the following variables are defined based on individual parameters:

```
r = 0.5 * (head_w / 2) #maximum distance from landmark
new_y = (eye_y + mouth_y) * 0.5 #mean distance, from nose to eyes and
    mouth
```

```
p1 = [-eye_x * 0.5, new_y] #left eye
p2 = [ eye_x * 0.5, new_y] #right eye
p3 = [          0.,-new_y] #mouth
dst_pts = np.array([p1, p2, p3]) #facial landmarks position
```

A function is then created to estimate the distance between two 2D positions:

```
def distance(pt, i): #distance formula
  return math.sqrt((i[0]-pt[0])**2+(i[1]-pt[1])**2)
```

The point of intersection can then be classified using the function expressed below:

```
def classify(pt,dst_pts,r):
  dps=np.array([distance(pt,i) for i in dst_pts]) #distance to the
   landmarks
  if np.min(dps)>r: #if the distance is further than r
    return 'outside'
  else:
    return ['left eye','right eye','mouth'][dps.argmin()]
```

References

Amblard, B., Assaiante, C., Lekhel, H., Marchand, A.R. (1994). A statistical approach to sensorimotor strategies: conjugate cross-correlations. *Journal of Motor Behavior*, *26*(2), 103-12. https://doi.org/10.1080/00222895.1994.9941665

Behrens, F., Moulder, R.G., Boker, S.M., Kret, M.E. (2020). Quantifying Physiological Synchrony through Windowed Cross-Correlation Analysis: Statistical and Theoretical Considerations. *bioRxiv*, 2020.08.27.269746. https://doi.org/10.1101/2020.08.27.269746

Bochkovskiy, A., Wang, C., Liao, H.M. (2020). YOLOv4: Optimal Speed and Accuracy of Object Detection. *CoRR*, *abs/2004.10934*. https://doi.org/10.48550/arXiv.2004.10934 https://arxiv.org/abs/2004.10934

Bradski, G. (2000). The OpenCV Library. *Dr. Dobb's Journal of Software Tools*.

Cornejo, C., Hurtado, E., Cuadros, Z., Torres-Araneda, A., Paredes, J., Olivares, H., . . . Robledo del Canto, J.-P. (2018). Dynamics of Simultaneous and Imitative Bodily Coordination in Trust and Distrust. *Frontiers in Psychology*, *9*, 1546. https://doi.org/10.3389/fpsyg.2018.01546

Derrick, T. (2004). *Time series analysis: The cross-correlation function.* Human Kinetics.

Hessels, R.S., Benjamins, J.S., Cornelissen, T.H.W., Hooge, I.T.C. (2018). A Validation of Automatically-Generated Areas-of-Interest in Videos of a Face for Eye-Tracking Research. *Frontiers in Psychology*, *9*. https://doi.org/10.3389/fpsyg.2018.01367

Labbe, R. (2014). *Kalman and bayesian filters in python.* GitHub. https://github.com/rlabbe/Kalman-and-Bayesian-Filters-in-Python

Ramseyer, F., and Tschacher, W. (2008). Synchrony in Dyadic Psychotherapy Sessions. , 329-347. https://doi.org/10.1142/9789812792426_0020

Reidsma, D., Nijholt, A., Tschacher, W., Ramseyer, F. (2010). Measuring Multimodal Synchrony for Human-Computer Interaction. *2010 international conference on cyberworlds* (p. 67-71). https://doi.org/10.1109/CW.2010.21

Schaller, U.M., Biscaldi, M., Burkhardt, A., Fleischhaker, C., Herbert, M., Isringhausen, A., ... Rauh, R. (2021). ADOS-Eye-Tracking: The Archimedean Point of View and Its Absence in Autism Spectrum Conditions. *Frontiers in Psychology*, *12*. https://doi.org/10.3389/fpsyg.2021.584537

Schoeneberger, J.A. (2016). The Impact of Sample Size and Other Factors When Estimating Multilevel Logistic Models. *The Journal of Experimental Education*, *84*(2), 373-397. https://doi.org/10.1080/00220973.2015.1027805

Sheerman-Chase, T. (2016). *Line-plane intersection.* https://gist.github.com/TimSC/8c25ca941d614bf48ebba6b473747d72. GitHub.

Sunday, D. (2021). *Practical geometry algorithms: With c++ code.* Amazon Digital Services LLC - KDP Print US. https://books.google.se/books?id=YBN0zgEACAAJ

Thorsson, M. (2022). *Face-to-face interaction analysis.* GitHub. https://github.com/thoraxmax/face-to-face-interaction-analysis

Virtanen, P., Gommers, R., Oliphant, T.E., Haberland, M., Reddy, T., Cournapeau, D., ... SciPy 1.0 Contributors (2020). SciPy 1.0: Fundamental

Algorithms for Scientific Computing in Python. *Nature Methods*, *17*, 261–272. https://doi.org/10.1038/s41592-019-0686-2
