# Supplementary material for: A novel end-to-end dual-camera system for eye gaze synchrony assessment in face-to-face interaction
Source: Atten Percept Psychophys. 2023 Apr 26;86(7):2221–30. doi: 10.3758/s13414-023-02679-4 (PMC11480169; doi:10.3758/s13414-023-02679-4)
Supplement: Supplementary file 2 — (PDF 281 KB) [file 13414_2023_2679_MOESM2_ESM.pdf]

Appendix B

Metrics to inform the performance of gaze estimation

M. Thorsson et al.

The supplementary text serves as an extended method to the paper, *A novel end-to-end dual-camera system for eye gaze synchrony assessment in face-to-face interaction*. Calculations of the metrics were based on the pre-processed data and condensed as mean or median (for median error) per participant/experimenter. The definition and mathematical equation for obtaining the metrics are described below. All metrics were estimated using Python (Rossum, 1995) 3.85 and the native functions of the NumPy library (Harris et al., 2020), version 1.20. All quality metrics were based on the estimated 2D position, at the intersection between the gaze vector and screen or the facial plane, and the distance from this point to the participant or experimenter (for estimation of degrees).

1 Gaze metrics

1.1 Robustness

We used robustness as the terminology for the percent of valid data (Vehlen et al., 2021). In other words, the amount of data that the system successfully captured. The loss of data can be due to several causes such as eyeblinks and head movement (Niehorster et al., 2018). Data is here excluded by a likelihood threshold from the Kalman filter, which is included in the robustness percentage. Robustness is here estimated using all recorded data points per participant.

1.2 Precision

This metric is describing the stability of the estimates and is here obtained from the standard deviation formula, as previously done by Vehlen et al. (2021) and recommended by Holmqvist et al. (2011). Standard deviation represents the deviation from the centroid (mean). A lower value indicates better precision. The equation for obtaining the precision is shown below.

Let $\overline{\mathrm{x}}$ and $\overline{y}$ be the coordinates of the centroid.

$$\text{Precision} = \sqrt{\frac{1}{\mathrm{n}} \sum_{i=1}^{n} (x_i - \overline{x})^2 + (y_i - \overline{y})^2}\ .$$

1.3 Median error

We define the median error as the median error in the distance (in degrees or cm), between the estimated position, on the monitor or facial plane, to the target position. First, all values are sorted by size. If the number of data points is even, the median is the value located at, $\frac{n}{2}$, where n is the number of data points. In contrast, if the number of data points is unequal it is the value at $\frac{n+1}{2}$, at the mean between the closest values. The median angular error has been reported for several deep learning-based eye tracking solutions (Rakhmatulin & Duchowski, 2020; Zdarsky et al., 2021). The median is a robust estimate of central tendency, as long as the outliers do not exceed 50% of the data.

1.4 Accuracy

The accuracy or operational accuracy is the average (mean) in degrees/cm to a target position that the participant looks at and is often used as a performance metric for eye tracking (Holmqvist et al., 2022). This is a measure of how close the estimate was to the position that the participant was expected to look at (here the calibration target on the screen or the target facial landmark located at the facial plane).

Let $\bar{a}_i$ be the mean gaze error (the total sum of all data points divided by the number of data points) per participant, then accuracy for all the participants can be obtained by the following equation.

$$Accuracy = \frac{1}{n}\sum_{i=1}^{n} \bar{a}_i \, .$$

2 Tables for gaze metrics

The following tables are displayed to provide further information about the gaze estimation performance, for Condition 3 in Experiment 1 and Experiment 2. See Table 1 for the metrics for gaze estimation during the screen calibration stimulus and Tables 2 and 3 for when verbally instructed to look at facial areas in the face-to-face experiment. The median error, accuracy, and precision were estimated for each event when the participant, respectively the experimenter, was expected to look at the target calibration point or facial area.

Table 1. Metrics for gaze at the 9-point calibration stimulus displayed on a monitor (n = 9, including experimenter, and experimenter alone in column farthest to the right, separated by the dotted line). Quality metrics were averaged across the gaze points per participant and experimenter.

	**min**	**max**	**mean**	**median**	**SD**	**Experimenter**
**Robustness**						
% valid data	80.01	97.72	91.89	93.21	6.00	90.71
**Precision (SD)**						
in degrees	0.90	1.50	1.14	1.15	0.19	0.92
in cm	1.23	1.99	1.55	1.60	0.24	1.60
**Median error**						
in degrees	0.98	2.74	1.90	2.04	0.50	2.74
in cm	1.30	4.81	2.62	2.56	0.92	4.81
**Accuracy**						
in degrees	1.41	3.22	2.15	2.09	0.56	3.22
in cm	1.92	5.57	2.98	2.84	1.04	5.57

Table 2. Metrics for gaze at facial areas (left and right eye and mouth) during verbal instructions in face-to-face interaction, for when the participants (n = 8) looked at the experimenter (n = 1). Quality metrics were averaged for the facial areas per participant.

	**min**	**max**	**mean**	**median**	**SD**
**Robustness**					
% valid data	72.19	93.51	87.41	89.52	6.29
**Precision (SD)**					
in degrees	1.61	7.73	3.67	3.22	1.88
in cm	1.03	5.64	2.34	1.77	1.43
**Median error**					
in degrees	1.54	6.13	3.13	2.51	1.59
in cm	1.63	6.65	3.42	2.74	1.80
**Accuracy**					
in degrees	2.03	9.23	4.25	3.07	2.47
in cm	1.75	9.59	4.09	3.15	2.68

Table 3. Metrics for gaze at facial areas (left and right eye and mouth) during verbal instructions in face-to-face interaction, for when the experimenter (n = 1) looked at the participants (n = 8). Quality metrics were averaged for the facial areas per participant.

	**min**	**max**	**mean**	**median**	**SD**
**Robustness**					
% valid data	87.83	94.45	91.64	92.81	2.30
**Precision (SD)**					
in degrees	1.44	3.49	2.16	2.02	0.66
in cm	0.85	2.13	1.31	1.31	0.37
**Median error**					
in degrees	1.25	5.65	2.71	2.48	1.35
in cm	1.33	6.06	2.92	2.84	1.37
**Accuracy**					
in degrees	1.50	6.80	3.22	2.81	1.62
in cm	1.44	7.12	3.18	2.88	1.65

References

Harris, C. R., Millman, K. J., van der Walt, S. J., Gommers, R., Virtanen, P., Cournapeau, D., . . . Oliphant, T. E. (2020). Array programming with NumPy. *Nature*, *585*(7825), 357-362. https://doi.org/10.1038/s41586-020-2649-2

Holmqvist, K., Nyström, M., Andersson, R., Dewhurst, R., Jarodzka, H., & van de Weijer, J. (2011). *Eye Tracking: A comprehensive guide to methods and measures*. OUP Oxford. https://books.google.se/books?id=5rIDPV1EoLUC

Holmqvist, K., Örbom, S. L., Hooge, I. T. C., Niehorster, D. C., Alexander, R. G., Andersson, R., . . . Hessels, R. S. (2022). Eye tracking: empirical foundations for a minimal reporting guideline. *Behavior Research Methods*. https://doi.org/10.3758/s13428-021-01762-8

Niehorster, D. C., Cornelissen, T. H. W., Holmqvist, K., Hooge, I. T. C., & Hessels, R. S. (2018). What to expect from your remote eye-tracker when participants are unrestrained. *Behavior Research Methods*, *50*(1), 213-227. https://doi.org/10.3758/s13428-017-0863-0

Rakhmatulin, I., & Duchowski, A. T. (2020). Deep Neural Networks for Low-Cost Eye Tracking. *Procedia Computer Science*, *176*, 685-694. https://doi.org/https://doi.org/10.1016/j.procs.2020.09.041

Rossum, G. v. (1995). Python tutorial. In: Centrum voor Wiskunde en Informatica Amsterdam.

Vehlen, A., Spenthof, I., Tönsing, D., Heinrichs, M., & Domes, G. (2021). Evaluation of an eye tracking setup for studying visual attention in face-to-face conversations. *Scientific Reports*, *11*(1), 2661. https://doi.org/10.1038/s41598-021-81987-x

Zdarsky, N., Treue, S., & Esghaei, M. (2021). A Deep Learning-Based Approach to Video-Based Eye Tracking for Human Psychophysics [Methods]. *Frontiers in Human Neuroscience*, *15*. https://doi.org/10.3389/fnhum.2021.685830
